# Environmental awareness and environmental information disclosure: An empirical study based on energy industry

**DOI:** 10.3389/fpsyg.2022.1038040

**Published:** 2022-11-21

**Authors:** Shijin Wang, Ziwen Zhou, Keqin Tian

**Affiliations:** ^1^Jiangsu Normal University, Xuzhou, China; ^2^University of Liverpool, Liverpool, GB, United Kingdom

**Keywords:** environmental awareness, environmental information disclosure, energy companies, structural equation model, environmental information disclosure quality

## Abstract

Environmental protection and governance have become a topic of global concern. Sustainable development and green development are the unshakable ideas of China’s social and economic development. Under this premise, the energy industry, as a pillar industry in China, has the disadvantages of high energy consumption and heavy pollution. Therefore, the quality of environmental information disclosure in energy industry needs to be improved urgently. This paper selects 66 samples from 22 energy companies in Shanghai and Shenzhen stock markets from 2018 to 2020 as the research object, establishes two potential variables and 13 observed variables of environmental awareness and environmental information disclosure respectively, and constructs structural equation model to study the impact of corporate environmental awareness on environmental information disclosure. The research shows that the environmental awareness of enterprises is positively related to the level of environmental information disclosure. The conclusion is helpful to improve the enthusiasm and consciousness of energy companies to disclose environmental information. A better role in supervising the quality of environmental information disclosure by the government, enterprises and the public can be played.

## Introduction

Since the reform and opening up, China’s economy has taken off rapidly, but at the same time it has also brought serious environmental problems. The important role of environmental information disclosure has gradually entered the public’s vision and has also been concerned by the state and the government. As a responsible country, China has vigorously promoted the construction of ecological civilization. In 2008, the State Environmental Protection Administration of the People’s Republic of China issued Decree No. 35, “Measures for the Disclosure of Environmental Information (for Trial Implementation),” which came into force. The purpose of the Decree is to explore the establishment of an environmental information disclosure system for Chinese enterprises, protect the stakeholders of enterprises and enable the public to obtain more comprehensive, open and transparent environmental information of enterprises. On June 12, 2017, the Ministry of Environmental Protection and the China Securities Regulatory Commission signed the Cooperation Agreement on Jointly Carrying out Environmental Information Disclosure of Listed Companies. The purpose of signing this agreement is to strengthen the environmental protection awareness of listed companies, supervise the environmental information disclosure of listed companies, realize the transparency and openness of environmental information disclosure of listed companies, enable the general public to more conveniently access environmental information, and make “Lucid waters and lush mountains are invaluable assets” a social reality.

Environmental awareness came into being in the western countries in 1960s. The reason why environmental consciousness can enter people’s consciousness field and gradually become a universal social consciousness in western society until today has its economic and social background. Under the influence of the third technological revolution, advanced science and technology were rapidly transformed into productive forces, industrial production in western countries increased at a high speed, and farmers’ production, gross national product and people’s living standards were greatly improved. However, due to the rapid development of industry, natural resources are constantly destroyed and wasted, the environment is deteriorating, the contradiction between man and nature is deepening, and people finally become victims of resource and environment problems (Si, 2021). Under the background, the concept of environmental awareness came into being. In 1992, the United Nations Conference on Environment and Development was a landmark event on the world’s environment and development issues. The conference released Agenda 21 and put forward the concept and initiative of “sustainable development.” According to the requirements of the United Nations, countries all over the world formulated their own sustainable development strategies and put them into practice. “Sustainable development” formally brings environment and development into a unified framework, forming a development concept of coordinated development and sustainable development, and begins to reflect on the traditional development concept of blindly pursuing development, neglecting or even seeking development at the expense of destroying the environment.

In summary, this paper selects 22 listed companies in the energy industry as samples, extracts the potential variables of environmental awareness and environmental information disclosure, and establishes a structural equation model to explore whether environmental awareness has an impact on environmental information disclosure.

## Literature review

The concept of environmental awareness emerged in the 1960s. It is a comprehensive concept that reflects many new problems in the relationship between human beings and the environment (Arcury, 1990), including five levels, especially knowledge, attitude, perceptual knowledge, evaluation and behavior. Liu Jianguo believes that environmental awareness refers to the sum of people’s understanding of the environment itself, the relationship between people and the environment and environmental protection. It is divided into two levels: rational knowledge, including thoughts, viewpoints, theories and perceptual knowledge, including psychology, emotion and attitude (Liu, 2008). China’s public environmental awareness is generally on the rise through a survey of China’s public environmental awareness level. It will enter a stable state in 2019 and the public environmental awareness is generally high (Liu et al., 2009; Yan et al., 2010). Huang provides information on the public’s perception of the local environmental quality, environmental awareness and environmental performance, and of their willingness to pay for improving environmental quality and making green purchases. The results indicate that residents are not satisfied with the local environmental quality, and they would like to share environmental responsibility (Huang et al., 2006).

There are two main explanations for the motivation of enterprises to disclose environmental information: socio-political theory and economic disclosure theory. Social and political theory covers a series of hypotheses to explain the disclosure behavior from the perspectives of legality, social system and stakeholders. It holds that companies passively disclose the environmental protection information of companies concerned by outsiders mainly based on the pressure of external information demanders, which indicates that companies have fulfilled the “social contract” to avoid threats such as legal lawsuits (Khan et al., 2013; Clementino and Perkins, 2021). Based on the signal transmission theory, the view is that companies voluntarily disclose environmental protection information to the outside world in order to obtain development resources, in order to show the company’s corporate image with good environmental performance, which is different from companies with poor performance and is recognized by the public. However, as the increasing relevance of Sustainable Development Goals in companies’ disclosure practices (Izzo et al., 2020), the government has gradually become the main body of environmental information disclosure (Yao, 2010; Wang et al., 2013). Shi Beibei pointed out that government environmental information disclosure can increase the public’s right to know and participate in environmental management of enterprises, improve the effect of environmental legislation and realize sustainable development (Shi et al., 2019). To sum up, environmental information disclosure refers to a kind of governance behavior in which the government and enterprises disclose relevant information about the environment in order to meet the supervision needs of the external public. The ultimate goal is to reduce corporate sewage discharge and realize sustainable economic development.

Scholars at home and abroad have not reached a consensus on the research index system of environmental information disclosure. From a comprehensive analysis of various aspects, factors such as enterprise size, industry, system level, financial status, proportion of senior executives holding shares, and audit level are generally accepted research influencing factors. The quality level of environmental information disclosure of listed companies in China is significantly related to company size, management expenses and policy implementation (Wang, 2019; Zhang, 2021). Wang Xinping made descriptive statistics on the data of more than 3,000 listed companies in Shanghai and Shenzhen. The results show that the perfection of the environmental information disclosure system of listed companies and the strengthening of the supervision of environmental violations have a significant positive impact on the degree of environmental information disclosure of listed companies (Wang and Li, 2021). Ortas processed and analyzed the collected data by quantile regression method. The results show that the indicators reflecting the financial position are significant factors that affect the disclosure of environmental information (Ortas et al., 2015). Environmental events, political relations, the nature of equity, barriers to entry have a significant impact on the environmental disclosure of heavily polluted listed companies (Luo and Lai, 2015). Li’s research results show that the greater the government’s supervision, the higher the level of corporate environmental information disclosure (Li, 2015). Carnini et al. think that there is a positive relationship between environmental, social, and governance disclosure and firm performance (Carnini Pulino et al., 2022). Another research shows that there is a significant positive correlation between environmental information disclosure and enterprise value and environmental cost (Wang et al., 2020). The results of Prasad show that factors such as corporate characteristics, size, age and foreign customers have significant positive impact on environmental disclosure, while leverage has negative impact on disclosure (Prasad et al., 2017). Chen explores the influencing factors and mechanisms of corporate environmental information disclosure. Through a random-effect GLS regression analysis of 363 listed manufacturing companies from the Shanghai Stock Exchange for 2012–2018, they find that the link between internal/external factors and environmental information disclosure is mediated by corporate environmental management (Chen et al., 2022). The factors affecting the quality of environmental information disclosure are very extensive, and the conclusions reached by most scholars using different research objects and methods are inconsistent, which need further research.

Xu Yongjie pointed out that environmental awareness and supervision are the driving force of environmental information disclosure (Xu, 2018). Xiao Hua conducted a research on environmental information disclosure and environmental awareness in China through a questionnaire survey. The survey results show that environmental information disclosure has been implemented in China’s enterprises at the present stage. Some enterprises are required to make mandatory disclosure, while others voluntarily disclose. However, there are still problems such as imperfect environmental accounting information system, incomplete environmental information disclosed, lack of comparability and reliability (Xiao, 2001). Chen shows that enterprises with strong environmental awareness have a higher level of environmental information disclosure. Exploring the relationship between them is helpful to improve the level of environmental information disclosure (Chen, 2016). To sum up, this paper establishes a structural equation model for corporate environmental information disclosure and environmental awareness to explore the relationship between environmental awareness and environmental information disclosure.

## Research assumptions

Enterprise performance will affect the environmental information disclosure behavior of enterprises to a certain extent. Generally speaking, the improvement of enterprise’s environmental performance will affect the enterprise’s awareness of environmental disclosure, enhance the enterprise’s awareness of environmental disclosure, and then the enterprise’s environmental information disclosure behavior will be better and better. However, there are some extreme situations, that is, the performance of enterprises is very poor, but the level of environmental information disclosure of enterprises is very good. This is because enterprises with poor environmental performance will be under the pressure of legitimacy and the pressure from the public, and their image and reputation are poor. In this case, in order to enhance their social reputation and gain recognition from stakeholders, enterprises will choose to disclose more environmental information to prove their efforts and achievements in the environment. Some scholars also randomly selected 300 households in Beijing and Guangzhou to conduct a questionnaire survey. The statistical results show that in China, although people pay more attention to ecological issues and hold positive ecological values, they cannot effectively implement them in their behaviors. Then enterprises with high environmental awareness do not necessarily apply this awareness to the environmental information disclosure of enterprises. Therefore, this paper puts forward the hypothesis:

*H*_0_: Environmental awareness negatively affects the level of environmental information disclosure.

Qingdao Haier Co., Ltd. has started voluntary environmental information disclosure since 2005. Its environmental information disclosure level is extremely high and the information disclosure content is detailed. Thus, when enterprises have higher environmental awareness, they pay more attention to environmental information and the environmental information disclosure level is also higher. However, it is also a non-heavily polluting enterprise. In 2014, Yanghe shares, respectively, exceeded the standard for the discharge of a number of pollutants, and twice appeared in the black list of pollutants released by the Jiangsu Provincial Environmental Protection Department. Yanghe shares had to disclose its own environmental information under the supervision of the government because it entered the field of vision of the Environmental Protection Department twice. In the case of repeated illegal discharge of pollutants, the public report of Yanghe shares did not disclose this matter. Obviously, the environmental awareness of this enterprise is relatively weak. However, the environmental information disclosed under such mandatory circumstances is lacking in content, with the intention of avoiding the environmental information that is unfavorable to itself. In summary, the following assumptions are made:

*H*_1_: Environmental awareness positively affects the level of environmental information disclosure.

## Research design

### Research framework

In this paper, SEM model is used for empirical research. Structural equation model is an analysis method based on statistical analysis technology. It combines the analysis of influencing factors with the related path analysis, and can be used to process and analyze the complex multivariable research data. SEM model can not only process and analyze complex data, but also estimate its potential variables and measure the model of complex variables. At the same time, the model itself allows measurement errors between independent variables and dependent variables. By establishing, estimating and testing causal relationship, SEM model has become a common and important statistical method in academic and professional research, which is applied in many professional fields such as economy, society and management.

There are three reasons for choosing these potential and observed variables. Firstly, this paper taken the standards of ISO (International Organization for Standardization), ISAR (International Standards of Accounting and Reporting), the Intergovernmental Working Group of Experts on International Standards and International Accounting and Reporting Standards. As well as sustainable development reports and social responsibility reports issued by listed energy companies. Secondly, the latest references are consulted to determine our variables. Thirdly, in view of the availability of data, variables from company are selected, including high-frequency indicators released by energy industry (Table 1).

**Table 1:** Summary of potential and observed variables.

Potential variable	Observed variable	Scoring rules
Environmental information disclosure	Wastewater discharge	1 for qualitative disclosure, 1 for quantitative disclosure and 0 for non-disclosure
	Sulphur dioxide emissions	
	Carbon dioxide emissions	
	Smoke and dust emissions	
	Industrial solid waste generation	
Environmental awareness	Separate disclosure of environmental report	1 for disclosure and 0 for non-disclosure
	Environmental objectives	1 for disclosure and 0 for non-disclosure
	Environmental education and training	1 for disclosure and 0 for non-disclosure
	Emergency mechanism for environmental events	1 for disclosure and 0 for non-disclosure
	Environmental protection management system	1 for disclosure and 0 for non-disclosure
	ISO14001 certification	Pass the score of 1, fail to pass the score of 0
	“Three Simultaneities” System	1 for disclosure and 0 for non-disclosure
	Environmental honors or awards	Get 1 point, not 0 point

### Determination of potential and observed variables

#### Data sources

According to the “Industry Classification Results of Listed Companies in the 4th Quarter of 2020” released by the China Securities Regulatory Commission, the energy companies mainly composed of oil, coal and natural gas are selected as samples for exploratory analysis. The selected sample basis follows the following principles: First, eliminating ST companies that will have certain impact on the research process; The second is to eliminate companies with too short listing time and incomplete information disclosure data; Third, the selection of A-share listed companies is beneficial to the consistency of statistical data. In the end, this paper selects a total of 66 samples from 22 energy companies in Shanghai and Shenzhen stock exchanges from 2018 to 2020. The basic information of the sample companies is shown in Table 2.

**Table 2:** Basic information of sample companies, statistics, stock codes, company abbreviations.

Stock code	Abbreviations of companies	Stock code	Abbreviations of companies
600028	Sinopec	600395	Panjiang Shares
600997	Kailuan Shares	000983	Shanxi Coking Coal
601088	China Shenhua	600759	Intercontinental Oil and Gas
300164	Tongyuan Oil	600348	Yangquan Coal Industry
600188	Yanzhou Coal Industry	603393	The Sky Is New
600123	Orchid Family Creation	601699	Lu 'an Huanneng
601898	China Coal Energy	601666	Pingmei Shares
601857	PetroChina Company Limited	600777	Trendy Energy
601225	Shaanxi Coal Industry	600758	Liaoning Energy
600256	Guanghui Energy	000937	Jizhong Energy
601001	Jincontrol Coal Industry	603689	Anhui Natural Gas

Juchao Information Network, Shanghai Stock Exchange, Shenzhen Stock Exchange.

#### Reliability and validity test

The reliability test of the questionnaire sample data is to ensure the accuracy and reliability of the questionnaire itself. If the internal standard of the sample data of the questionnaire is uniform, the quality of the questionnaire can be considered to be better, and further analysis can be carried out. Cronbach’s α is often used to measure the internal quality of questionnaire sample data. From Table 3, we can see that Cronbach’s *α* of the scale is above 0.7, from which we can draw the following conclusion: the sample data of the questionnaire in this paper have passed the sample reliability test and meet the standard. At the same time, this article tries to do the test again after deleting the items. After deleting, the value of Cronbach’s α has not changed greatly. This shows that the reliability level of the measurement items set in this paper is high.

**Table 3:** Reliability test of the scale.

Potential variable	Observed variable	Cronbach’s alpha after item deleted	Cronbach’s *α*
Environmental consciousness	Environmental statement	0.759	0.778
	Environmental objectives	0.734	
	Environmental education and training	0.717	
	Emergency mechanism for environmental events	0.765	
	Environmental protection management system	0.751	
	ISO14001 certification	0.784	
	“Three Simultaneities” System	0.771	
	Environmental honors or awards	0.741	
Environmental information disclosure	Wastewater discharge	0.703	0.809
	Sulphur dioxide emissions	0.688	
	Carbon dioxide emissions	0.820	
	Smoke and dust emissions	0.746	
	Industrial solid waste generation	0.840	

Next, this paper conducts confirmatory factor analysis (CFA) on the environmental awareness and environmental information disclosure of the latent variables, and uses AMOS26.0 to conduct CFA on the scale. Fit the constructed confirmatory factor model and observe whether the model construction is accurate by combining with the fitting index. See Table 4 for the results of CFA. CR is greater than 0.7, which proves that the measurement items can better interpret the corresponding potential variables. That is, the five observed variables can consistently explain the potential variable of environmental information disclosure. Eight observed variables can consistently explain the potential variable of environmental awareness.

**Table 4:** Validation factor results.

Path			Normalized factor load	Nonstandard factor load	S.E.	*t*-value	*p*	SMC	CR	AVE
Environmental information disclosure	<−--	Environmental consciousness	0.807	1.607	0.572	2.808	0.005			
CO2 emissions	<−--	Environmental information disclosure	0.485	1				0.235	0.819	0.505
SO2 emissions	<−--	Environmental information disclosure	0.915	2.748	0.695	3.955	***	0.837		
Wastewater discharge	<−--	Environmental information disclosure	0.825	2.044	0.535	3.822	***	0.681		
Smoke and dust emissions	<−--	Environmental information disclosure	0.824	2.245	0.6	3.744	***	0.679		
Industrial solid waste generation	<−--	Environmental information disclosure	0.302	0.428	0.209	2.05	0.04	0.091		
environmental statement	<−--	environmental consciousness	0.539	1				0.291	0.786	0.326
Environmental objectives	<−--	environmental consciousness	0.629	2.01	0.559	3.594	***	0.396		
Environmental education and training	<−--	environmental consciousness	0.791	2.348	0.569	4.128	***	0.626		
Emergency mechanism for environmental events	<−--	environmental consciousness	0.461	1.486	0.524	2.834	0.005	0.213		
Environmental protection management system	<−--	environmental consciousness	0.584	1.764	0.533	3.312	***	0.341		
ISO14001 certification	<−--	environmental consciousness	0.347	0.713	0.309	2.31	0.021	0.120		
“Three Simultaneities” System	<−--	environmental consciousness	0.456	1.362	0.483	2.819	0.005	0.208		
Environmental honors or awards	<−--	environmental consciousness	0.642	1.691	0.454	3.727	***	0.412		

*** Significant at 99% significance level.

### Model validation and hypothesis testing

**Figure 1:**
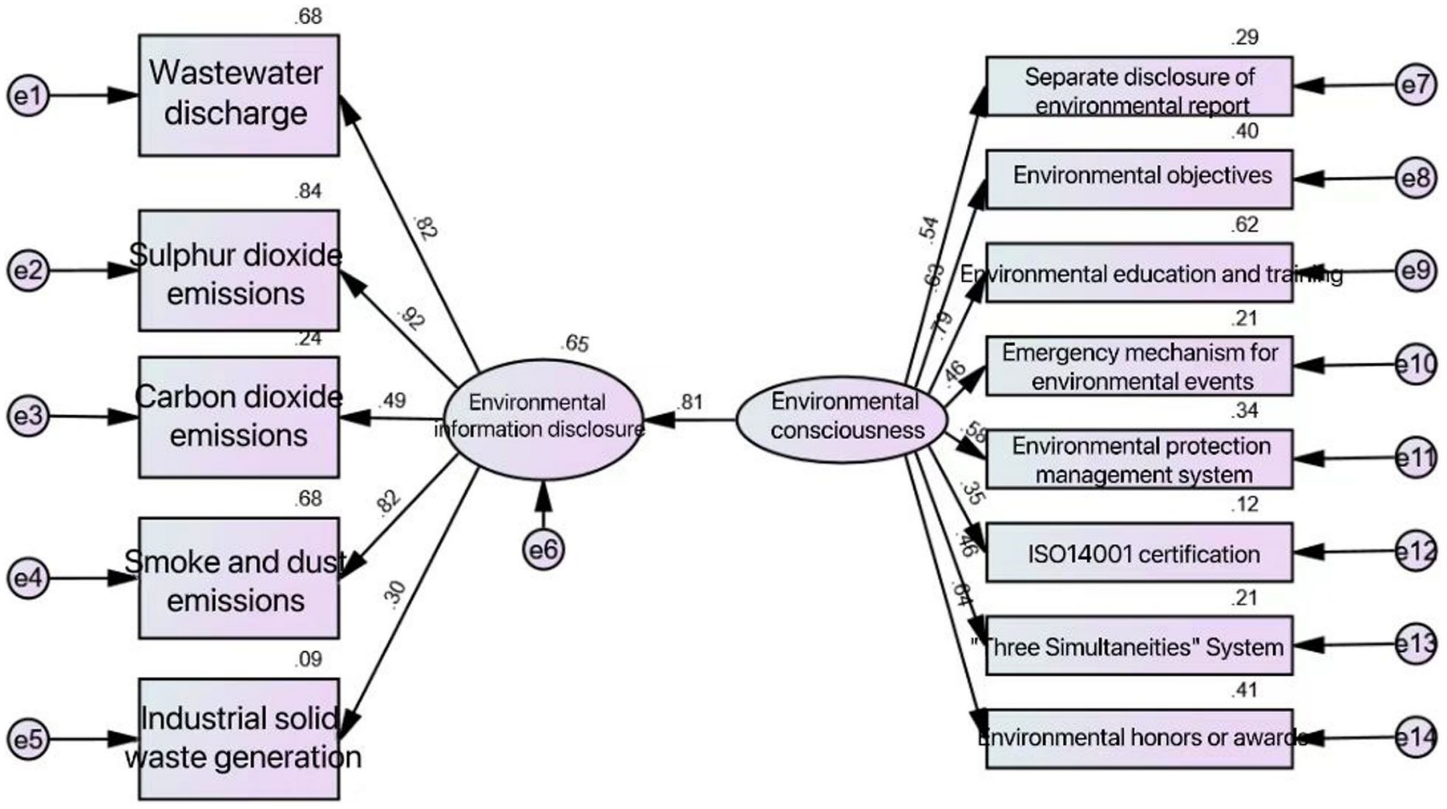
AMOS output results.

Using the structural equation model, the final model shown in Figure 1 is obtained. The indicators of the model are Chi-square/df and GFI. Generally, Chi-square/df needs to be between 1 and 3. If it exceeds 3, the fitting is not good. If it is less than 1, the fitting is excessive. The best value for both GFI is close to 10.8. Chi-square is 152.3, df is 64, Chi-square/df = 2.38, Chi-square is less than 3 degrees of freedom; the GFI value is 0.733, close to 0.8, which meets the general standard. The overall fitting degree of this model is good, and the internal quality of the model reaches the standard.

Firstly, the standardized factor load is observed. The standardized factor load of environmental awareness on environmental information disclosure is 0.807, which is very important. The importance of wastewater discharge, sulfur dioxide discharge, carbon dioxide discharge, smoke dust and dust discharge and industrial solid waste production to environmental information disclosure is 0.82, 0.92, 0.49, 0.82 and 0.30, indicating that the disclosure of indicators such as wastewater discharge, sulfur dioxide discharge, carbon dioxide discharge, smoke dust and dust discharge and industrial solid waste production can positively measure the environmental information disclosure, in which the impact of wastewater discharge, sulfur dioxide discharge, smoke dust and dust discharge is stronger; The impact coefficients of environmental report, environmental protection objectives, environmental protection education and training, environmental event emergency response mechanism, environmental protection management system, ISO14001 certification, “three simultaneities” system, environmental honor or award items on environmental awareness are 0.29, 0.40, 0.62, 0.21, 0.34, 0.12, 0.21 and 0.41, indicating that their impact on environmental awareness is positive, among which the impact degree of environmental protection education and training is strong, while the impact degree of environmental report, environmental event emergency response mechanism, ISO14001 certification and “three simultaneities” system is weak.

From the data of non-standardized factor load, the non-standardized factor load of environmental awareness on environmental information disclosure is 1.607, with a *p* value of 0.005, indicating that environmental awareness and environmental information disclosure are significant at 95% significance level, which proves that *H*_1_, environmental awareness has significant positive impact on the level of environmental information disclosure. Denying *H*_0_, environmental awareness has a negative impact on the level of environmental information disclosure. It shows that environmental awareness has a positive impact on environmental information disclosure, and a better environmental awareness is beneficial to enterprises to improve the level of environmental information disclosure.

## Conclusion

This paper mainly studies its impact on the level of environmental information disclosure from the perspective of environmental awareness. Based on the above empirical analysis, the following conclusions are drawn: the higher the environmental awareness of the enterprise, the higher the level of environmental information disclosure. The level of environmental awareness of an enterprise will have an important impact on whether the enterprise performs its environmental responsibilities. For companies, companies with a high degree of awareness pay more attention to the performance of their environmental responsibilities and correspondingly improve the quality and initiative of environmental information disclosure. Therefore, it is of great significance to improve the environmental awareness of enterprises to improve the level of environmental information disclosure.

### Implications

This study provides some enlightenment for the managers. Managers need to pay attention to the correct combination of companies images and environmental awareness. If the enterprise shows a positive environmental awareness, it can highlight the “green value” of the products, thus improving consumers’ loyalty. High-quality environmental information disclosure may lead to the increase of negative information of enterprises in the short term, but in the long run, it will help enterprises regulate their own market behavior, improve their social responsibility awareness and strengthen the public supervision, thus helping enterprises to establish a good corporate image and enhance their value.

This research is of great significance for the sustainability of the energy industry. After consulting the literature, environmental sustainability regards different research areas (logistics, supply chain management, and transportation; innovation management; information systems; engineering; Centobelli et al., 2017). It can also be combined with digital transition (Abbate et al., 2022). It’s an inevitable trend for the energy industry to cross with environmentally sustainable development (Huber and Hirsch, 2017).

### Limitation and future research direction

Firstly, the data collection year span is short, which limits the industrial and timeliness. Expanding the sample size and extending the time span can help the model improve its persuasiveness. With the development of economy, the number of listed companies is increasing, and the relevant data will be updated constantly, so as to expand the sample size and improve the credibility of the model.

Secondly, the selection of observation variables in this paper has certain limitations. For example, the questionnaire survey can be used to bring the individual’s inherent concept and behavior of environmental protection into the model as variables. Moreover, some variables play intermediary roles between environmental awareness and environmental information disclosure, such as digital cross technology and green transformation technology. These factors that may affect the experimental results have not been taken into account, which is the deficiency of the model. Further research can be started from these sides.

## Data availability statement

The datasets presented in this study can be found in online repositories. The names of the repository/repositories and accession number(s) can be found in the article/Supplementary material.

## Author contributions

SW is the architect and the person in charge of the project, guiding the experimental design, data analysis. ZZ is the experimental designer and executor of the study, who completes the data analysis. The drafting and revision opinions are completed by KT.

## Funding

This study was supported by grants from the National Social Science Foundation of China, “Research on the mechanism and path of collaborative management of haze pollution in China from the perspective of regional linkage” (nos. 19BGL196); Jiangsu University Advantageous Discipline Construction Project (PAPD); Jiangsu Provincial Social Science Excellent Youth Project in 2019; Jiangsu Provincial Youth and Blue Project in 2019 Funded project; Carbon Dafeng Carbon Neutral Technology Innovation Special Incubation Program of Jiangsu Normal University in 2022.

## Conflict of interest

The authors declare that the research was conducted in the absence of any commercial or financial relationships that could be construed as a potential conflict of interest.

## Publisher’s note

All claims expressed in this article are solely those of the authors and do not necessarily represent those of their affiliated organizations, or those of the publisher, the editors and the reviewers. Any product that may be evaluated in this article, or claim that may be made by its manufacturer, is not guaranteed or endorsed by the publisher.
